# Systematic reduction of natural enemies and competition across variable precipitation approximates buffelgrass invasiveness (*Cenchrus ciliaris*) in its native range

**DOI:** 10.1002/ece3.11350

**Published:** 2024-05-11

**Authors:** Aaron C. Rhodes, Robert M. Plowes, Elizabeth A. Bowman, Aimee Gaitho, Ivy Ng'Iru, Dino J. Martins, Lawrence E. Gilbert

**Affiliations:** ^1^ Brackenridge Field Laboratory The University of Texas at Austin Austin Texas USA; ^2^ Hiro Technologies, Inc Austin Texas USA; ^3^ Mpala Research Centre Nanyuki Nanyuki Kenya; ^4^ Turkana Basin Institute Nairobi Kenya; ^5^ UK Centre for Ecology & Hydrology Cardiff University Wallingford UK

**Keywords:** insect herbivory, invasiveness, natural enemies, pathogens, plant competition, ungulate herbivory

## Abstract

Invasive grasses cause devastating losses to biodiversity and ecosystem function directly and indirectly by altering ecosystem processes. Escape from natural enemies, plant–plant competition, and variable resource availability provide frameworks for understanding invasion. However, we lack a clear understanding of how natural stressors interact in their native range to regulate invasiveness. In this study, we reduced diverse guilds of natural enemies and plant competitors of the highly invasive buffelgrass across a precipitation gradient throughout major climatic shifts in Laikipia, Kenya. To do this, we used a long‐term ungulate exclosure experiment design across a precipitation gradient with nested treatments that (1) reduced plant competition through clipping, (2) reduced insects through systemic insecticide, and (3) reduced fungal associates through fungicide application. Additionally, we measured the interaction of ungulates on two stem‐boring insect species feeding on buffelgrass. Finally, we measured a multiyear smut fungus outbreak. Our findings suggest that buffelgrass exhibits invasive qualities when released from a diverse group of natural stressors in its native range. We show natural enemies interact with precipitation to alter buffelgrass productivity patterns. In addition, interspecific plant competition decreased the basal area of buffelgrass, suggesting that biotic resistance mediates buffelgrass dominance in the home range. Surprisingly, systemic insecticides and fungicides did not impact buffelgrass production or reproduction, perhaps because other guilds filled the niche space in these highly diverse systems. For example, in the absence of ungulates, we showed an increase in host‐specific stem‐galling insects, where these insects compensated for reduced ungulate use. Finally, we documented a smut outbreak in 2020 and 2021, corresponding to highly variable precipitation patterns caused by a shifting Indian Ocean Dipole. In conclusion, we observed how reducing natural enemies and competitors and certain interactions increased properties related to buffelgrass invasiveness.

## Graphical abstract

This study conducted in Laikipia, Kenya, investigates the impact of reducing natural enemies and plant competitors on the invasiveness of buffelgrass, a highly invasive species. The researchers employed a long‐term ungulate exclosure experiment across a precipitation gradient with treatments that targeted plant competition, insects, and fungal associates. The findings suggest that buffelgrass exhibits invasive qualities when released from diverse natural stressors, with precipitation and natural enemies interacting to influence its productivity.

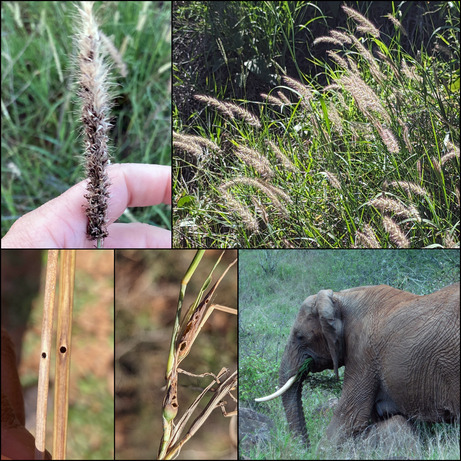

## 1 INTRODUCTION

Invasive grasses are a leading cause of biodiversity loss and ecosystem degradation worldwide (Canavan et al., 2019; D'Antonio & Vitousek, 1992). Anthropogenic redistribution of exotic forage grasses for rangeland development has broad historical practices (Cox et al., 1988; Parsons, 1972) and remains a major goal of many agroeconomies (Jank et al., 2014; Sukhchain., 2010). Despite acknowledging the detriment to native flora and fauna, economic drivers promote human‐mediated land use change and subsequent grass invasion (Driscoll et al., 2014; Sukhchain., 2010). Invasive grasses are implicated in changing fire regimes that contribute to the degradation of ecosystems from the Mojave Desert (Brooks et al., 2004) to the Amazon Rainforest (Cochrane et al., 2009) and loss of life and property (Rains, 2023). Given the urgent challenges of invasive grasses, examining their invasiveness is necessary. Yet, our understanding of invasion ecology is complicated by the milieu of contributing biotic and abiotic factors, including natural enemies, plant–plant competition, precipitation, and climate.

The interconnectedness of these biotic and abiotic invasion factors likely precludes simple invasion conceptual models or simple management solutions. A multitude of hypotheses describe single mechanisms of invasion ecology and help describe the natural phenomena (Jeschke et al., 2012); however, many are redundant and interconnected (Catford et al., 2009). The redundancy likely reflects the complex factors that promote or inhibit invasion. Biotic and abiotic interactions can filter invasion by limiting the ability of novel organisms to invade novel environments (Cleland et al., 2013). The interactions also alter organismal functional traits through the founder effect and genetic drift during establishment (Eckert et al., 1996). These filters can determine initial success and naturalization through biotic resistance via competition or consumption and disease from native organisms (Kennedy et al., 2002). For instance, abiotic filters determine invasibility through the availability of resources (Ricciardi et al., 2021), and disturbances reduce biotic resistance, release resources, or both (Davis et al., 2000; Maron et al., 2013). While release from a specific enemy can explain invasion potential (Torchin et al., 2003), many biotic and abiotic interactions likely determine an organism's ability to invade (Catford et al., 2009). When multiple guilds of natural enemies are considered, their relative impact on invasive plants may shift (Agrawal et al., 2005) and vary across resources and plant communities' diversity (Heckman et al., 2017). A common experimental technique compares invasive species' productivity and reproductivity in their native and invaded ranges (Maron et al., 2013; Taylor et al., 2016); however, processes used to describe between ranges—propagule pressure, escape from many natural enemies and stressors, novel weapons, and an evolved competitive advantage (Blossey & Notzold, 1995; Callaway et al., 2022; Hierro & Callaway, 2003; Keane & Crawley, 2002; Torchin et al., 2003)—likely occur in concert, adding to the complexities of interpreting invasion (Blumenthal, 2006; Heckman et al., 2016). Therefore, a holistic approach may lead to insights into how these processes occur simultaneously and impact invasion success. Here, we simulated the biotic conditions that an invasive grass would face outside of its native range, by experimentally reducing natural enemies of different taxa and interspecific plant competitors, with the aim of disentangle their impacts and interactions across variable precipitation and climate.

The dangers of climate change to Earth's ecosystem are well‐studied and continue having wide‐ranging impacts (IPCC, 2023) that will undoubtedly exacerbate grass invasion. A changing climate potentially increases invasiveness (Ricciardi et al., 2021) and invasibility (Chambers & Pellant, 2008). Biological invasion can progress along variable environmental gradients (Wang et al., 2019), and resource fluctuations driven by episodic weather extremes often open up niches for invading organisms (Davis et al., 2000; Zhang et al., 2023). Many invasive species may perform better under variable precipitation regimes (Bradley et al., 2010; Ricciardi et al., 2021); however, grass distributions expand or contract based on their specific physiology (Williams & Baruch, 2000) and interactions with the biological communities within their respective niche space (Lopes et al., 2023). A possible mechanism is that invasive grass traits related to high propagule pressure, high fecundity, germination, and establishment may provide high adaptability to variable climates (Bradley et al., 2010; Simberloff, 2009). However, variable precipitation is expected to alter plants, their herbivores, and microbial associates in complex ways (Dukes et al., 2009). Understanding how invasive grass under variable precipitation interacts with other biotic filters of invasion, such as natural enemies and competitors, will provide insights into how invasions may proceed.

Among natural enemies, ungulates have shaped the evolution of grasses through direct consumption and indirectly through their impact on ecosystem processes (Augustine & McNaughton, 1998). As such, grasses introduced into new ranges may have higher invasive potential if they escape from regions of high abundance and diversity of ungulates (Rhodes, Plowes, Martins, et al., 2022). In the grass' native range, ungulates likely reduce properties such as resource acquisition, biomass storage, and seed production related to invasiveness, and the management use of generalist ungulates to replace this loss can be seen in the success of some targeted grazing programs. Targeted grazing has effectively reduced invasive grasses and increased species richness (Marchetto et al., 2021), indicating a potential escape from higher herbivory rates in their native range. Ungulates have differential impacts on plant communities that change long‐term vegetation characteristics (Goheen et al., 2018; Louthan et al., 2019), which may reduce the ability of highly competitive palatable grasses to dominate a plant community, as they do when invading novel habitats. By the same logic, releasing an invasive grass from ungulates in its native range should promote a competitive advantage over other plants and approximate an invasion scenario.

Higher interspecific competitive ability of nonnative grasses occurs by direct resource competition, apparent (indirect) competition through a loss of natural enemies, and an evolved competitive ability in the absence of native stressors (Callaway et al., 2022; Gioria & Osborne, 2014). Enhanced competitive ability of invasive grasses results in higher growth rates and higher nutrient turnover and can be driven by novel weapons and changes in soil biomes and chemistry (Bowman et al., 2023; Morrison, Rhodes, et al., 2023). This improved productivity also leads to higher propagule pressure and spread. Vegetation structure and competition outcomes often depend on abiotic resources and herbivory (Fine et al., 2004; Goheen et al., 2018), and the magnitude and direction of competitive effects between invaders and native plants may also shift across environmental gradients (Alpert et al., 2000; Lucero et al., 2020). Without natural enemies, production and reproduction may increase (Callaway et al., 2022). However, the competitive advantage of an invasive plant may not necessarily be solely due to its competitive edge and is likely conflated with its escape from enemies, alterations to soil properties, or allelopathy (Gioria & Osborne, 2014). Thus, field experimental observations of such invasives in their home ranges facilitate understanding plant–plant competition in the context of native enemies such as herbivores and pathogens.

Insect herbivores can drive plant community assembly directly through consumption or indirectly through apparent competition and plant resource availability (Endara et al., 2023; Fine et al., 2004). Insects interact with resource availability such that herbivory may increase in high‐resource environments relative to low‐resource environments, which may have downstream impacts on invasiveness (Li et al., 2022). Equally, insect communities compete and interact in complex ways to alter the productivity and reproduction of plants differentially (Agrawal et al., 2005). African grasses may lose a suite of these natural enemies in their introduced range (Morrison, Plowes, et al., 2023; Rhodes, Plowes, Martins, et al., 2022), contributing to their invasiveness. Additionally, using host‐specific insects to control grass invasion has shown success (Goolsby et al., 2011; Schwarzländer et al., 2018; Sutton et al., 2019). Therefore, we assume that reducing the impact of associated insect herbivores should approximate the invasion of plants in their native range.

Plant‐fungi relationships can promote invasive processes, yet they act in complex networks along a continuum of positive to negative interactions (Dickie et al., 2017). Fungal associates can have substantial top‐down impacts on plant performance and reproduction (Dean et al., 2012; Sun et al., 2020), and altering these associates may increase plant invasiveness (Mitchell & Power, 2003). Some endophytic fungi may be beneficial through improved herbivory defenses and environmental tolerances (Bamisile et al., 2018). Soil biota in native ranges can suppress invasion and may escape deleterious pathogens in the introduced ranges (Maron et al., 2014). Reducing fungal pathogens has been shown to increase the invasiveness of exotic plants (Heckman et al., 2016). However, pathogens of native African grasses are often understudied (Farrell et al., 2002), and thus assigning an ecological function to specific fungi is a complex and ongoing process (Nguyen et al., 2016). In this study, we assume that reducing fungal endophytes in their native range and measuring their impact on the host plant can improve our understanding of the importance of fungi in regulating plant invasions.

To understand the many biotic and abiotic factors that interact to mediate invasion, we designed an experiment that quantified the effects of precipitation, herbivory by ungulates, arthropods, and fungal associates on the invasiveness of buffelgrass (*Cenchrus ciliaris* (L.) Link) in its native range. Our goal was to reduce biotic and abiotic filters suppressing a native grass in its native range to explore key factors associated with its invasive potential in introduced ranges. We hypothesized that ungulate herbivory, competition with plants, insect herbivory, and fungal pathogens negatively impacted buffelgrass basal area, height, and reproduction. In addition, we hypothesized that there were interactive effects with precipitation and associations with weather shifts governed by the Indian Ocean Dipole. We tested these hypotheses by excluding ungulates, removing competing plants, and applying systemic insecticides and fungicides. We measured the impact that ungulates had on two likely buffelgrass‐specific insects: a galling wasp, (Hymenoptera: Eurytomidae: *Tetramesa* sp.) and a gall midge (Diptera: Cecidomyiidae: *Orseolia* sp.; Morrison, Plowes, et al., 2023) and hypothesized that ungulates reduced insect herbivory. Finally, we tracked fungal damage and observed an outbreak from seed smut (Ustilaginomycetes) impacting buffelgrass after a shift in extremes of the Indian Ocean Dipole. Examining the impact of these highly specialized insects presents an opportunity to understand their potential for shifting resource use by abundant and diverse guilds of natural enemies. This experimental and observational framework offers multifaceted approaches to reducing ecological stressors of buffelgrass in its native range in order to understand grass invasiveness.

## 2 MATERIALS AND METHODS

### 2.1 Study organism

**FIGURE 1:**
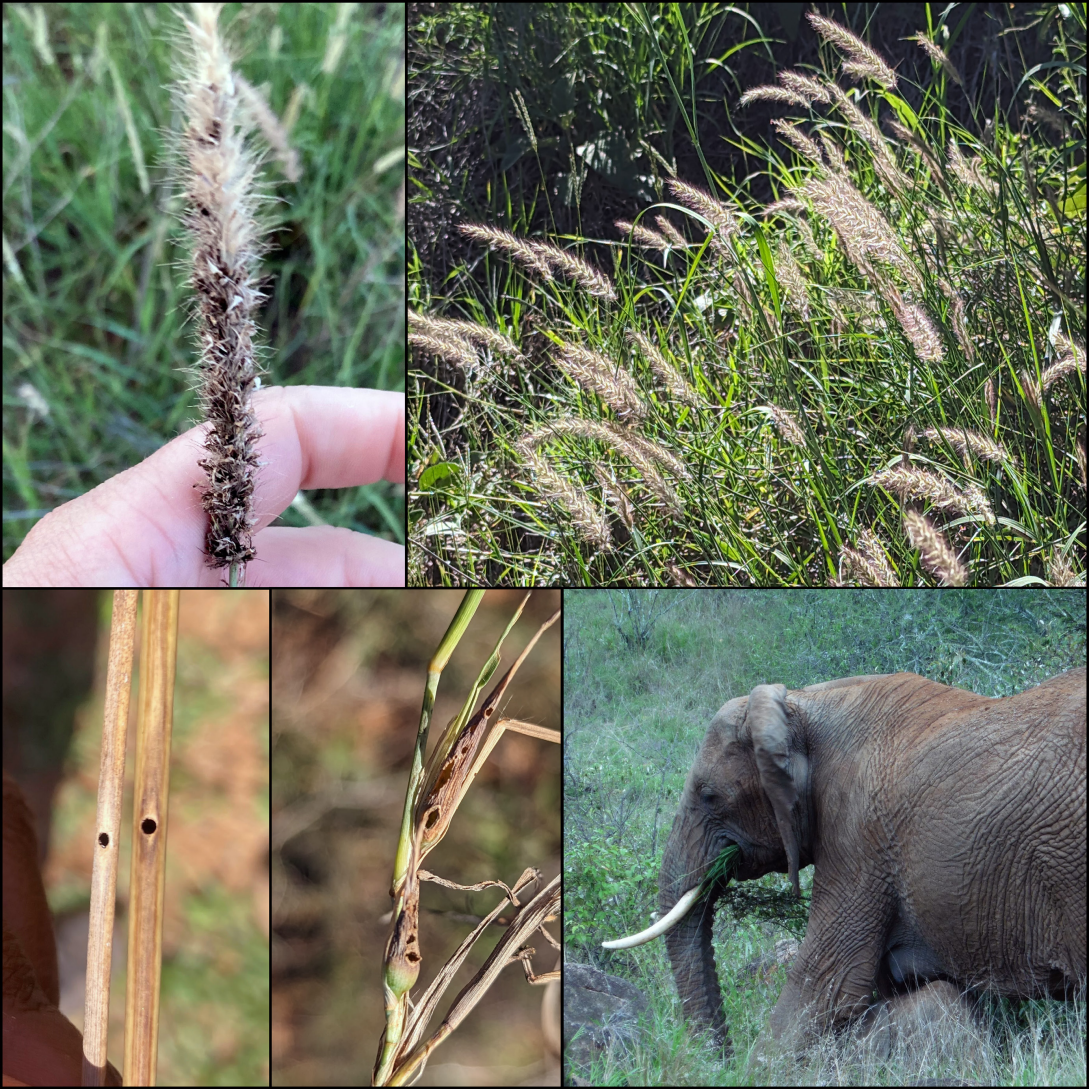
Buffelgrass associates: Top left: Buffelgrass seed head with fungal smut infection. Top right: healthy stand of buffelgrass. Bottom left: Stem gall exit holes generated by Tetramesa sp. stem‐galling wasp (L) and other insect (probably a beetle) (R). Bottom center: Galls with exit holes made by Orseolia sp. stem‐galling midges. Bottom right: Elephant grazing on grass.

Buffelgrass is native to Africa, portions of the Middle East, and India, but pantropically introduced. Buffelgrass is a tall‐statured, C4 perennial grass species utilized for livestock forage development that regularly escapes cultivation and becomes an ecologically damaging invader (Marshall et al., 2012). Like many introduced African grass species, it outcompetes other plants through water and nutrient‐efficient C4 photosynthetic pathways (Sage, 2004), high growth, and high reproductive rates (Canavan et al., 2019). Buffelgrass escapes multiple natural enemies from its native range (Figure 1), likely contributing to its competitive success and spread. These natural enemies include an abundant and diverse insect community that utilizes living and dead tissues (Morrison, Plowes, et al., 2023). Invading buffelgrass reduces native plant and wildlife diversity (Fulbright et al., 2013) and increases wildfire frequency and intensity, reducing non‐fire‐adapted native communities (McDonald & McPherson, 2011). Buffelgrass displays competitive advantages in its introduced range from drought tolerance, herbivory tolerance, and pathogen resistance, all contributing to high productivity and reproductivity (Marshall et al., 2012). Alarmingly, buffelgrass is likely to continue expanding under future climate projections (Ravi et al., 2022) due to its dynamic response to highly variable precipitation (Rhodes et al., 2023). Our study was predicated on the idea that examining the characteristics of buffelgrass invasion through productivity and propagule pressure under different abiotic and biotic conditions in its native range would improve the understanding necessary for integrated invasive grass management in its introduced range.

### 2.2 Study site

**FIGURE 2:**
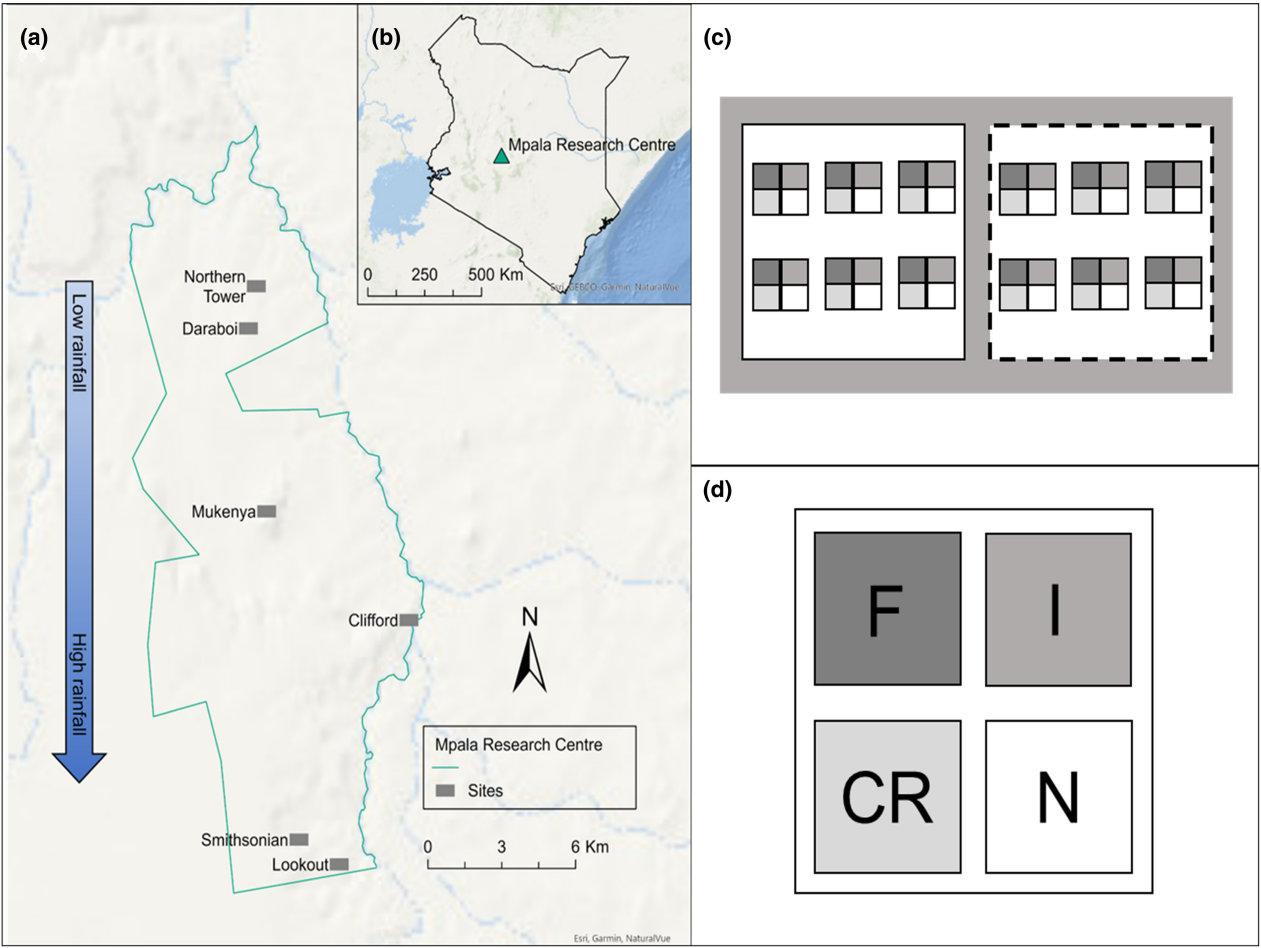
(a) Mpala Research Centre has six study locations along a precipitation gradient of 400 to 600 mm. (b) Mpala Research Centre shown in an inset map of Kenya's political boundaries. (c) A fenced and an unfenced plot are shown adjacent. Each gray box above the dotted line represents one of six randomized blocks across the 50 m by 50 m plot. (d) A zoomed‐in depiction of the randomized block with the four treatment subplots labeled as F – Fungicide, N – Control, I – Insecticide, and CR – competition release. Subplots were randomly arranged and spaced within each block.

The study was conducted at the Mpala Research Centre, part of a private conservancy in Laikipia County, central Kenya (0.292° N, 36.896° E). Mpala Research Centre is a research facility and working ranch situated along the Ewaso Ng'iro River in a semi‐arid tropical savanna in the rain shadow of Mt. Kenya. The Centre is 20,000 hectares, surrounded by local community lands, large ranches, and conservancies. The savanna vegetation is characterized by dominant trees/shrubs: *Senegalia mellifera* (Benth.) Seigler & Ebinger*., S. brevispica* (Harms) Seigler & Ebinger, *Vachellia etbaica* (Schweinf.) Kyal. & Boatwr., *Croton* spp. and diverse grasses *Cenchrus ciliaris, Digitaria mianjiana, Cynodon dactylon, Pennisetum stramineum*. The long axis of Mpala (30 km) stretches across a rainfall gradient with a 50% increase from lower (400 mm) to higher (600 mm) precipitation (Figure 2a). Six sites along this precipitation gradient were selected, two each in northern, central, and southern regions across Mpala. We conducted our research in sites on red sand soils (15% clay and 74% sand) and deep transition soils, well‐drained sandy clay loam to clay loam (Ahn & Geiger, 1987).

### 2.3 Ungulate associates

Mpala Research Centre manages a herd of approximately 3000 livestock grazing at low to moderate stocking intensities, including Zebu/Boran mix‐breed cattle, camels, goat, and sheep. Livestock grazing is conducted via traditional pastoralism centered on bomas (overnight holding pens) that are moved after several months but are haphazardly used every few years, leaving a distinctive patch of fertile soil and a radiating gradient of grazing pressure (Augustine, 2004; Goheen et al., 2018). In addition, formal arrangements allow communal grazing on Mpala alongside the ranch livestock. Mpala Research Centre regularly records 18 ungulate species (Rhodes, Plowes, Martins, et al., 2022; Riginos et al., 2012). In order of abundance in observations from camera traps at the site; *Bos taurus* Linnaeus, 1758*, Equus quagga* Boddaert 1785*, Aepyceros melampus* Lichtenstein 1812*, Loxodonta africana* Blumenbach 1797, *Giraffa camelopardalis reticulata* De Winton 1899, *Syncerus caffer* Sparrman 1779, *Equus grevyi* Oustalet 1882, *Ovis aries* Linnaeus 1758, *Madoqua guentheri* Thomas 1894, *Kobus ellipsiprymnus* Ogilby 1833, *Tragelaphus strepsiceros* Pallas 1766, *Taurotragus oryx* Pallas 1766, *Phacochoerus africanus* Gmelin 1788, *Capra hircus* Linnaeus 1758, *Nanger granti* Brooke 1872, *Raphicerus campestris* Thunberg 1811, and *Hippopotamus amphibius* Linnaeus, 1758. Wild ungulates move freely across the wider Laikipia ecosystem with variable seasonal patterns (Crego et al., 2020, 2021).

### 2.4 Climate and weather

Bimodal rainfall influenced by onshore monsoons characterizes precipitation patterns in Laikipia County called the “long rains” from March to May and the less wet and shorter duration “short rains” from October to December (Camberlin & Wairoto, 1997). The 30‐year average (1991–2021) rainfall for the 3‐month long and 3‐month short rains at Mpala Research Centre headquarters was 269 ± 24 mm and 145 ± 17 mm, respectively (Caylor et al., 2022). During the study period, various Indian Ocean Dipole phases increased the variability of precipitation and temperatures in Kenya. Negative Indian Ocean Dipole phases, associated with drier conditions, occurred in July 2021 through the end of 2021 and July through October 2022. The phase was neutral otherwise (AGBM, 2022).

### 2.5 Reduction of natural stressors in subplots

In February 2018, six sites were selected, each with two 50 x 50 m adjacent plots with similar topographic features and abundances of buffelgrass. One was randomly selected to exclude all ungulates, and the other was opened to ungulate herbivory. The fences were constructed by May 2018. Exclusion fences were erected 2.4 m tall, with wooden support poles in the corners and metal wire panel fencing. Seven high tensile wires ran along the wooden poles: five live wires carrying electric current and two dead wires spaced 0.3 m apart. A 0.8 m‐high chicken mesh further reinforced the perimeter of each exclosure to deter small animals. These exclusion fences were constructed with 3 m tall wooden poles at each corner, with nine additional poles 5 m apart.

The fences were electrified with solar‐powered electric fencing, maintaining a pulsed electrical voltage using fence controllers. This system included a 100 W Solinc East Africa Ltd. solar panel (1 x 0.7 m) mounted atop a 1.1 x 0.8 m power box. Inside the power box, a Hammer Energizer EZ 630 (Hammer Energizer, Johannesburg, South Africa) with a 4 J, 9 kV output capacity ensured sufficient voltage for the fence, while a 60 A Chloride Solar charge controller and a Chloride Exide 100/12/Solar maintenance‐free battery (Chloride Exide Kenya Ltd, Nairobi, Kenya).

We established six paired sites with two sites each at southern, central, and northern locations (Figure 2a). In June 2018, at each of the six sites, six replicates of four treatments were set up as 1 × 1 m subplots in a random block design in both fenced and unfenced plots, for a total of 288 subplots (Figure 2c,d). The subplot corners were marked with two 1 cm diameter 0.5 m tall iron reinforcing bars (rebar) set into the ground at opposite corners of the plot, and a numbered aluminum tag was attached to one corner rebar. Subplots were selected to include the focal species buffelgrass with a beginning abundance of at least 10% basal area cover and three tussocks in each subplot. Basal area is a cross‐sectional measurement that defines the area occupied by the perennial buffelgrass tufts. Subplots were at least 2 m apart from each other. In each subplot, a treatment was imposed for the duration of the study. We assigned to each subplot a treatment: (1) control, (2) interspecific competition release, (3) insecticide, and (4) fungicide. In June 2018, the treatments were initiated, and the first survey was completed in February 2019.

The competition release consisted of clipping all non‐buffelgrass biomass at the soil surface once per season after the surveys. To reduce insect damage to buffelgrass, insecticide was applied directly onto buffelgrass tussocks in their subplots at the onset of rains each long or short rain season (October or November and April or May, respectively) and repeated weekly throughout the active growing season. We used Actara 25WG (Basel Switzerland, Syngenta AG), a systemic broad‐spectrum insecticide that controls Aphididae, Cicadellidae, Thripidae, and Aleyrodidae, with thiamethoxam as the active ingredient. The dose was 0.02 g/m^2^, made by mixing 0.5 g per 2.5 L and applied by mist spraying on leaves.

To reduce fungi on buffelgrass, a systemic fungicide was applied directly onto buffelgrass tussocks in their subplot at the start of the active growing season and repeated 1 week later. The application was repeated each month through the rainy seasons. The fungicide used was Ridomil Gold MZ 68WG (Syngenta AG, Basel, Switzerland). This broad‐spectrum systemic chemical controls for oomycete diseases such as root rot, late blight, and downy mildew with metalaxyl‐M 40 g/kg and Mancozeb 640 g/kg as the active ingredients. The chemical is a wettable granule applied per the manufacturer's suggestions. The dose was 0.25 g/m^2^, made by mixing 6.25 g per 2.5 L and applied by mist spraying on leaves and a soil drench. Control and competition release subplots received an application of 100 mL/m^2^ of water whenever the insecticide and fungicide treatments were applied. We found that this volume was appropriate to achieve full coverage of the area and scale the needed dosage well without adding excess water to the experiment.

### 2.6 Data collection

#### 2.6.1 Buffelgrass productivity and reproduction and the plant community

To evaluate buffelgrass productivity, we measured buffelgrass basal area and height. We chose the two largest buffelgrass tussocks for each subplot, recorded the basal circumference to the nearest 1 cm, and calculated the area in cm^2^. The height of the tallest leaf was recorded in centimeters. We measured reproductive output by counting the number of inflorescences. We averaged these metrics for the largest two buffelgrass tufts to represent the buffelgrass productivity of the subplot. To estimate the diversity, we calculated the Shannon Diversity of the plant community; we recorded all species' identities and foliar cover to the nearest 0.1%. Additional cover types were litter and bare ground. The subplots were surveyed twice yearly from 2019 to 2022, following peak primary productivity periods associated with Kenya's “long rains” (March to May) and “short rains” (October to December) seasons.

#### 2.6.2 Insect specialists

We performed an additional experiment across the six sites to quantify the impact of specialist insects and their interaction with ungulates. Groups of buffelgrass tussocks were treated with insecticide (as above) every 2 weeks for 6 weeks from May to June of 2023 inside and outside the ungulate fencing. The control treatments were sprayed with an equal volume of water (100 mL/m^2^). At the end of the 6 weeks, the tussocks were harvested to identify *Orseolia* sp. (Cecidomyiidae) galls and exit holes of *Tetramesa* sp. wasps (Eurytomidae); both were previously shown to be frequent herbivores of buffelgrass (Morrison, Plowes, et al., 2023). At each site, inside and outside the fencing, an average of 50 ± 5 tillers were randomly harvested from each insecticide and control plot, for a total of 1238 tillers. The number of tillers per plot and exit holes was assessed and then divided into likely *Tetramesa* sp. and not, based on the morphology of the exit holes. Exit holes with characteristic symmetric circle anatomy with smooth edges were considered *Tetramesa* sp. and holes with asymmetry and jagged edges were classified as other insects (Borror & DeLong, 1971; Figure 1). Stem galls created by *Orseolia* sp. were counted.

#### 2.6.3 Fungal smut survey

Beginning in June 2019, we categorized smut, rusts, and blights (Ustilaginomycetes, Pucciniomycetes, and Oomycetes, respectively) by visually inspecting leaves within each subplot. Preliminary fungal disease analyses showed no patterns across subplot treatments. During these surveys, we observed a low smut incidence across buffelgrass samples in 2019. However, in July 2020, an outbreak of smut occurred on the developing seeds of buffelgrass. Using our broad survey from 2019, we surveyed specifically for the smut on the reproductive tillers in 2020, 2021, and 2022. In addition, we measured the absence–presence data of the smut at the subplot treatment level in 2020 and 2021. Preliminary smut analysis showed that the treatments and the interaction between year and treatments were not significant. This suggests that clipping, or spraying with insecticide or fungicide did not impact smut incidence, so future surveys looked at control subplots only. Beginning 2020, we measured annually the total number of seed heads for the two largest bufflegrass plants in each plot and the number of seed heads on the same two individuals with signs of a smut infection. Infection was defined as the appearance of black spore masses in all spikelets of an inflorescence (Kabaktepe et al., 2016; Figure 1).

To place the smut phylogenetically, we collected smut spores. Following the manufacturer's instructions, we extracted total genomic DNA from the spores using the RedExtract‐N‐Amp plant PCR kit (Sigma‐Aldrich, St. Louis, Missouri, USA). The internal transcribed spacer region (ITSrDNA) and portions of the adjacent nuclear ribosomal small subunit (SSU) and large subunit (SSU) were PCR amplified using primers smITS‐F and smITS‐R1 following Piątek et al. (2015). Sequence comparisons with NCBI and UNITE libraries indicated that the causal agent in the 2020 Kenya outbreak likely belonged to *Anthracocystis* sp. (Altschul et al., 1990).

To place our strain phylogenetically within *Anthracocystis*, we pulled sequences from Piątek et al. (2015). Using Sanger sequencing, we extracted the SSU, ITS1, ITS2, and LSU regions with ITSx (Bengtsson‐Palme et al., 2013) and concatenated them. We aligned the concatenated sequences with MAFFT (ver. 7; Katoh et al., 2019) using the default settings. We created a maximum likelihood tree with trimmed and aligned sequences in MegaX (ver. 10.2.4; Stecher et al., 2020). Taxonomic identification of the smut sequence was conducted through GenBank querying and validated using UNITE to help identify the pathogen behind the smut outbreak in Kenya.

### 2.7 Statistical analyses

Buffelgrass basal area, height, reproduction, and Shannon's Diversity were analyzed using mixed‐effects linear regression using the package nlme in R (Pinheiro et al., 2012; R Core Team, 2021). The assumptions of normality and homoscedasticity were assessed by visually comparing a histogram of model residuals and a plot of standardized residuals versus predicted values, respectively. The basal area was first analyzed as the square root of the circumference of buffelgrass measured in the field and then converted to area for graphical display. Reproductive tillers were not normally distributed, so we transformed the data by log(*x* + 1). Heteroskedasticity was modeled using weights of a variance identity structure by survey year. Outliers were assessed using Cleveland dot plots (Cleveland, 1993). Plots were used as a random variable in the mixed‐effects analyses to account for repeated measures. Site location (north, central, or southern) is used as a fixed factor to help account for spatial autocorrelation. To assess autocorrelation visually, we plotted the autocorrelation function (ACF). We found that lag decayed well for all response variables. Further, we added ARMA autocorrelation structures to the models, but they did not improve the ACF. For each response variable, we built a model that included years (2019, 2020, 2021, 2022), season (long or short rains), fence (open/close), treatment (control, clipping, insecticide, fungicide), and accumulated 6 months of precipitation. The interactions between treatment and precipitation, fence and year, and the interaction between fence and precipitation were further included. We used maximum likelihood to decide which interaction term was significant and dropped insignificant terms before reporting the final results (Zuur et al., 2009). Predicted values from each model were utilized for graphical representation purposes. Specifically, the parameter estimate for precipitation was employed to calculate its effect at a given site and year. Then, differences in treatment effects were displayed. For example, mean precipitation values within each year at each location were used to estimate predicted variables for treatment and fence effects.

To assess ungulates' effect on buffelgrass damage by specialist insects, we used a full‐factorial linear model to assess the main effects of ungulate access, systemic insecticide, and their interaction. The number of *Orseolia* sp. galls and *Tetramesa* sp. exit holes per study site for each experimental unit was used as the response variable. The north, central, and southern regions were used as categorical variables. Normality and heterogeneity of variance were assessed by inspecting residual plots.

Smut data, collected as presence–absence, were analyzed using logistic regression. A subplot was scored as having an absent or present infection (1 or 0). Subplots with no reproductive tillers were excluded from the analysis. A logistical regression with treatment, precipitation, year, reproductive tillers, and their interactions was run on the binomial distribution. We used maximum likelihood to decide which interaction term was significant. The log‐odds were converted to probability for interpretation.

## 3 RESULTS

### 3.1 Overview

Buffelgrass responded to a release from multiple natural enemies across a precipitation gradient by excluding ungulates and removing competing plants, but not by applying systematic insecticide and fungicide to reduce insects and fungi. Overall, precipitation and ungulate herbivory interacted to be the largest drivers of buffelgrass productivity and reproduction. The removal of competition increased buffelgrass productivity and tended to increase reproductive output. While applying insecticide and fungicide did not have significant results at the subplot scale, we saw that host‐specific insects increased with ungulate removal. We observed a precipitation‐associated smut outbreak that caused broad reductions in buffelgrass reproduction. Overall, our results showed that a release from several ecological stressors in the native range increased buffelgrass dominance and reduced plant community diversity, similar to what has been observed in its introduced range (Rhodes et al., 2021, 2023).

### 3.2 Ungulates, precipitation, and subplot treatments effects on buffelgrass and diversity

**FIGURE 3:**
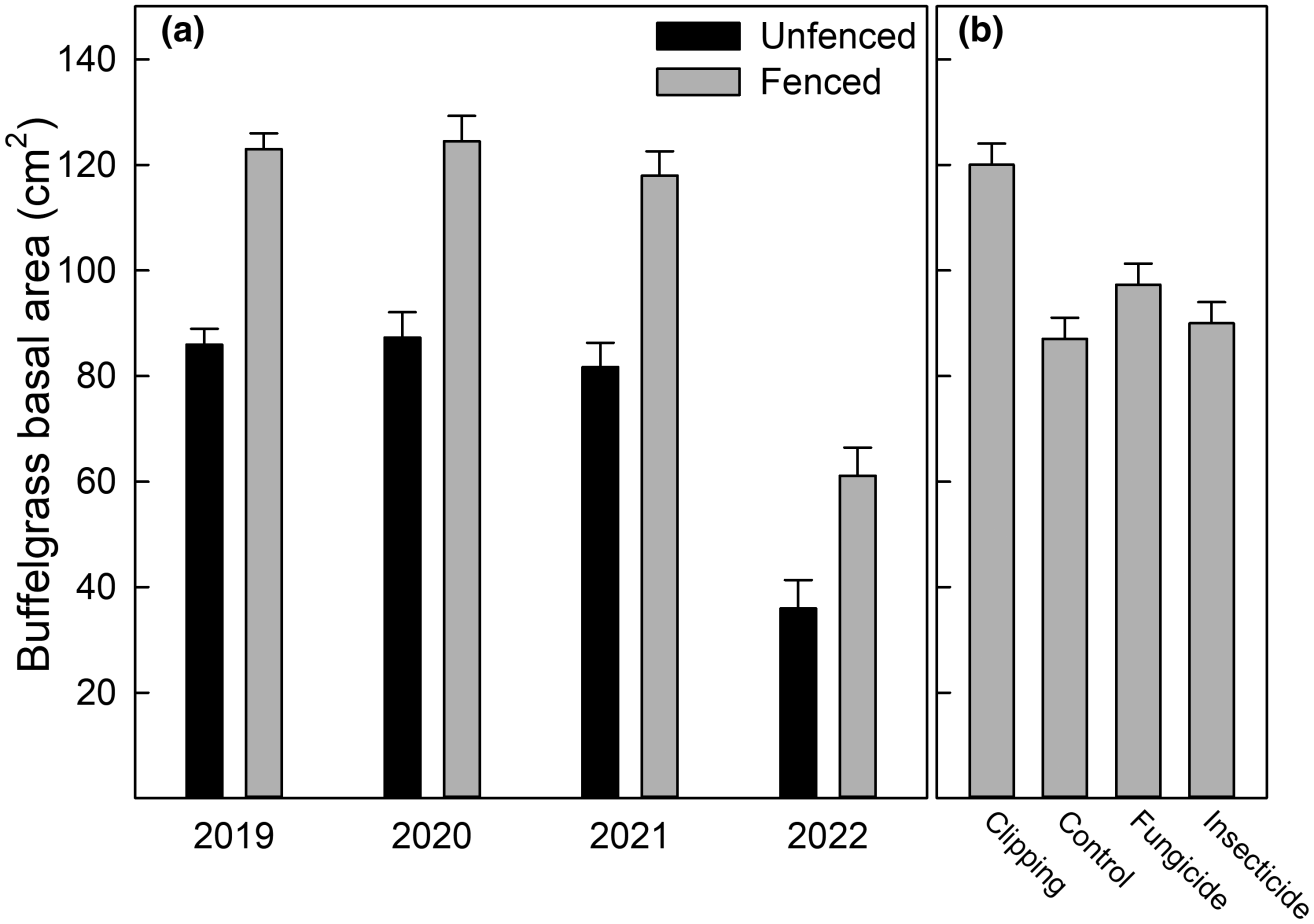
(a) Buffelgrass basal area as a function of fenced and unfenced plots is shown across the study years. (b) The effect of the treatments is displayed on the same scale as panel (a). The error bars for each panel represent one standard error.

**FIGURE 4:**
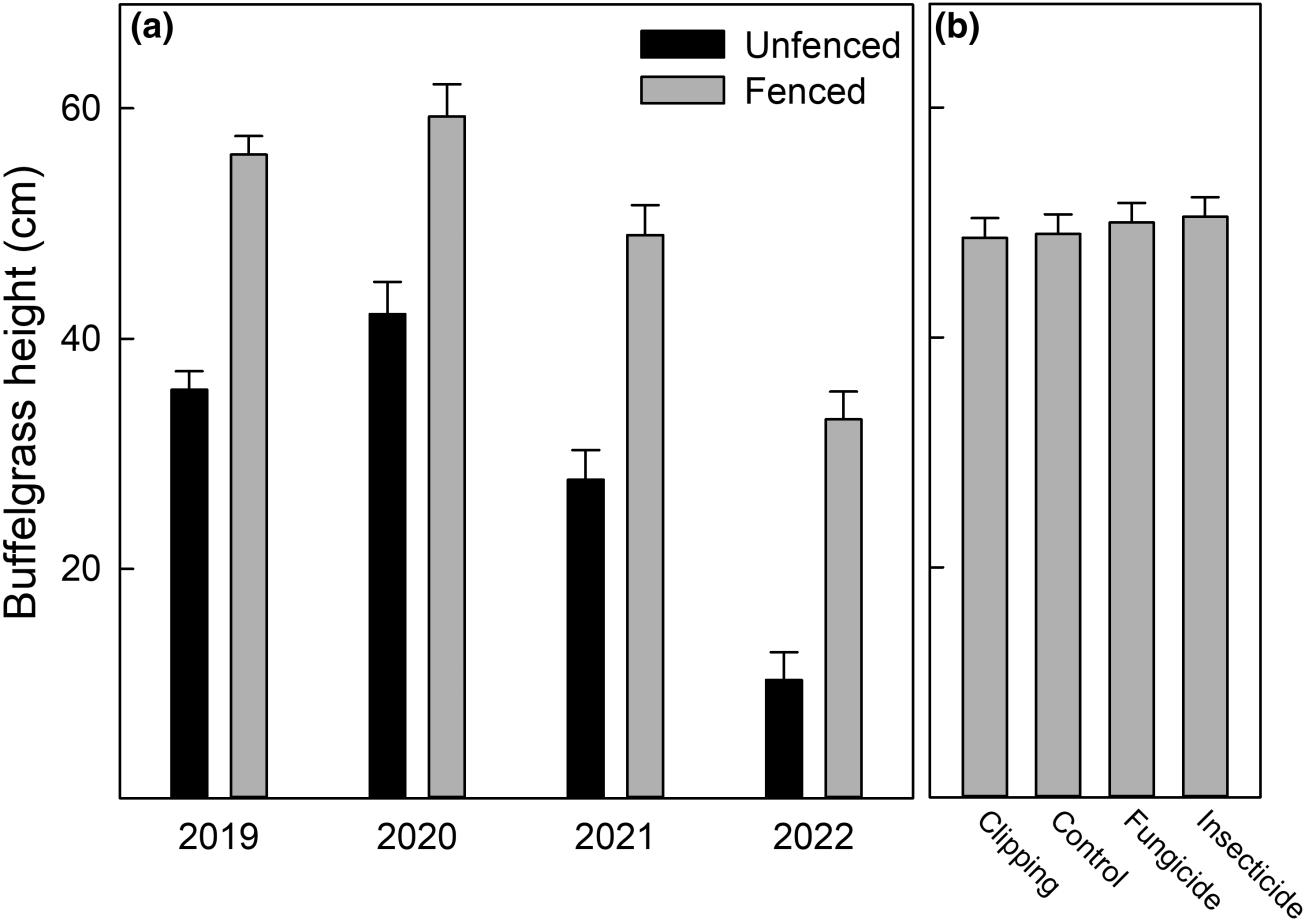
(a) Buffelgrass height is shown as a function of fenced and unfenced plots across the study years. (b) The effect of the treatments is displayed on the same scale as panel (a). The error bars for each panel represent one standard error.

**FIGURE 5:**
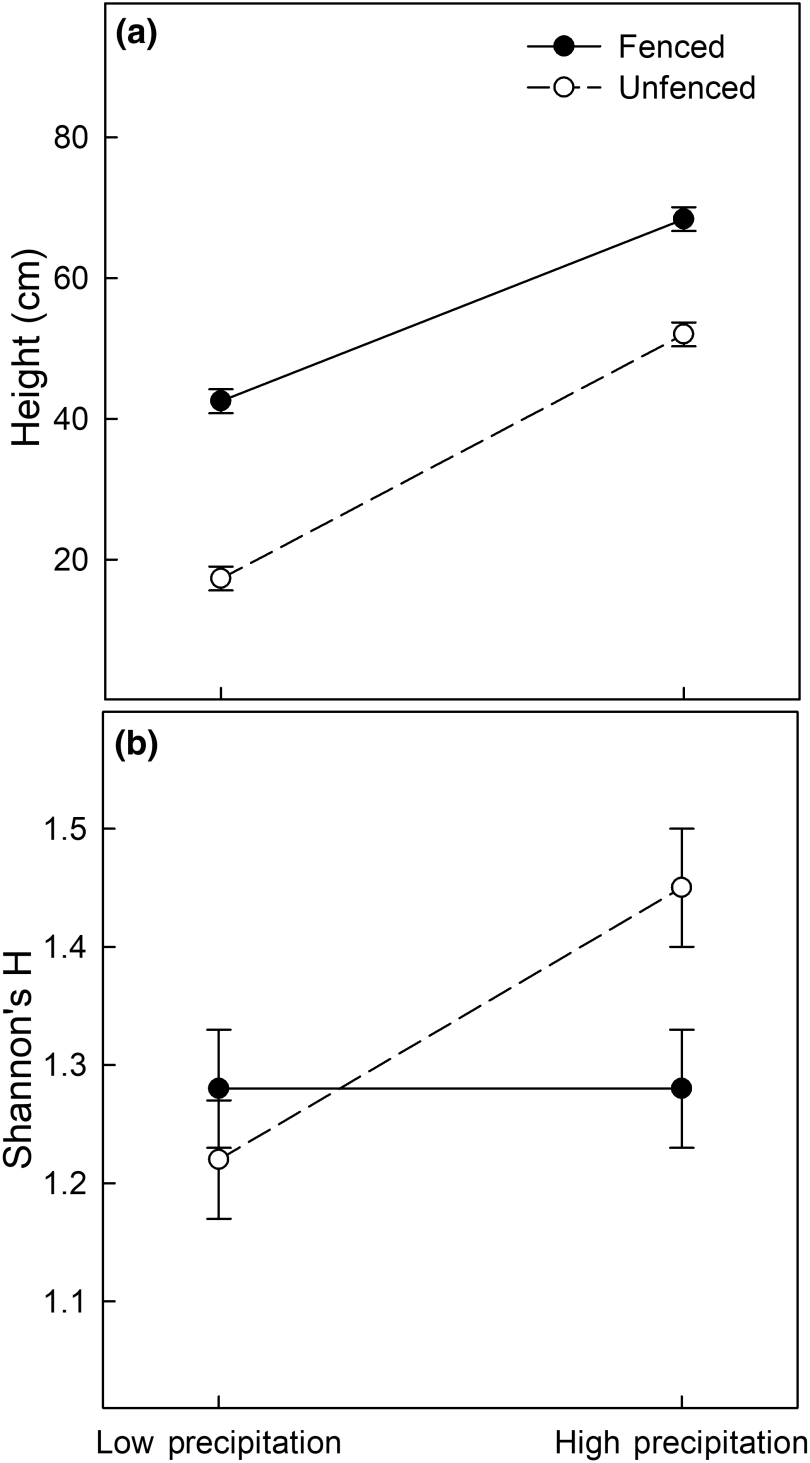
Interaction plots showing the model estimated height at the low lag precipitation (100 mm) and high lag precipitation (560 mm) range. Fenced plots are denoted with a solid line and filled circles, while the unfenced plots are denoted with a dashed line and open circles. The standard error is shown with the error bars. All other parameters are held at their mean and height (a) and Shannon's H (b) are shown in two panels.

Ungulate fencing and subplot treatments altered the direction and magnitude of drivers affecting buffelgrass production and reproduction. Precipitation and ungulate herbivory were the strongest factors influencing buffelgrass basal area (*F* = 17.8, *p* < .001, *F* = 18.7, *p* < .001, respectively) and buffelgrass height (*F* = 900, *p* < .001, *F* = 303, *p* < .001, respectively). There was no interactive effect of precipitation and fencing on buffelgrass basal area (Figure 3a). The basal area of buffelgrass increased when competing plants were clipped (*F* = 4.0, *p* = .008; Figure 3b). Buffelgrass height was impacted by ungulate herbivory, precipitation, and their interaction (Figure 4a), but was not significantly impacted by treatments of clipping fungicide and insecticide (Figure 4b). Precipitation and ungulate herbivory interacted such that ungulate herbivory contributed to a stronger relative height reduction in periods of higher precipitation (*F* = 28.5, *p* < .001; Figure 5a).

**FIGURE 6:**
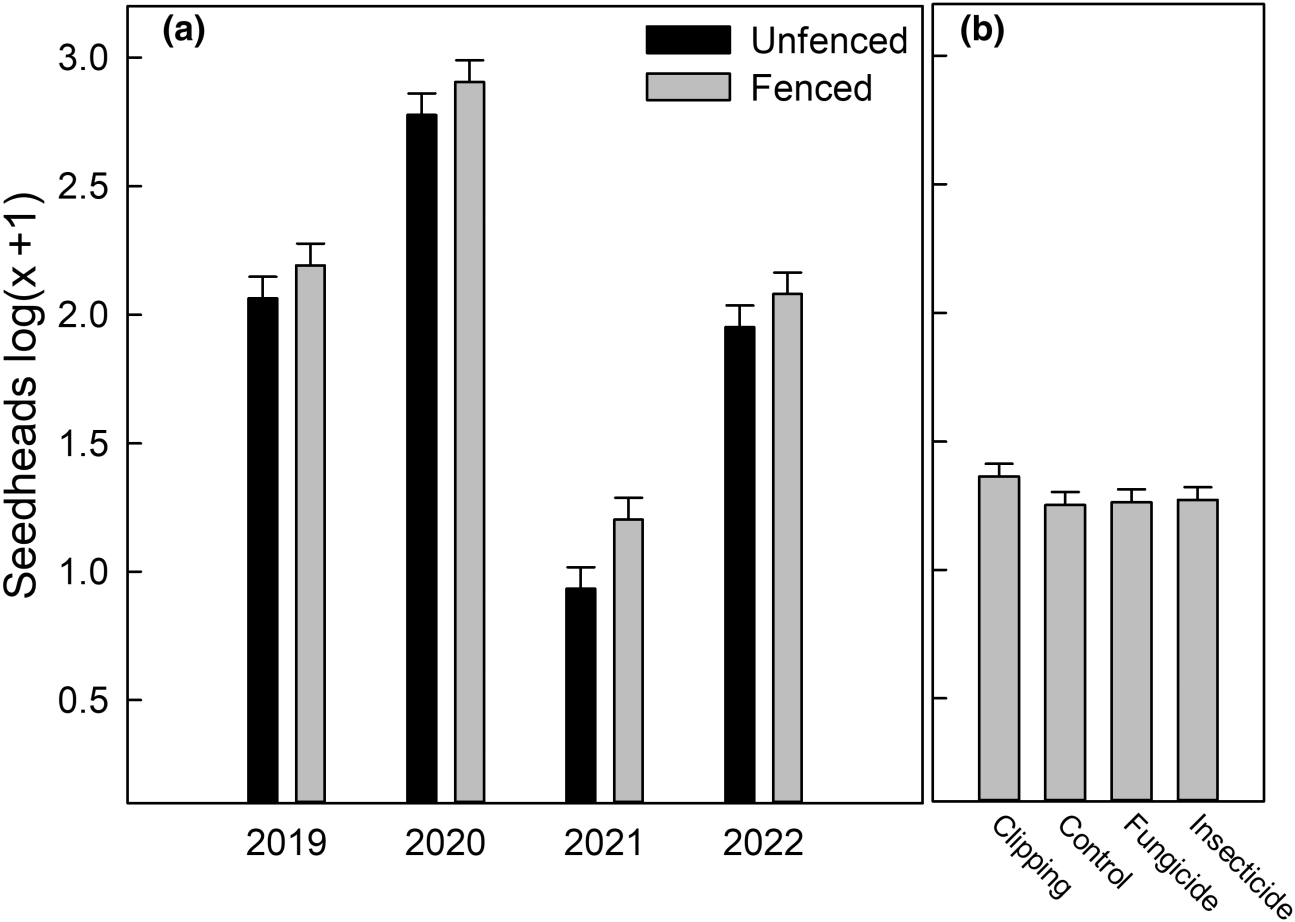
(a) The mean reproductive tiller count per tussock is shown as a function of fenced and unfenced plots across the study years. (b) The effect of the treatments is displayed on the same scale as panel (a). The error bars for each panel represent one standard error.

Competition tended to reduce reproductive tillers, but reproduction followed a large variation in precipitation, making rainfall the strongest predictor of reproduction. Ungulate herbivory significantly impacted the mean number of reproductive tillers per tussock, with slightly fewer reproductive tillers outside as opposed to inside the fence (*F* = 13, *p* < .001; Figure 6). Anecdotally, short buffelgrass would still produce a similar number of small tillers. Precipitation (*F* = 810, *p* < .001) and year (*F* = 447, *p* < .001) drove significant changes in reproductive tillers. Reduced competition by clipping tended to produce a higher number of buffelgrass seedheads. Still, the effect was not statistically significant (*F* = 1.9, *p* = .12; Figure 6b), perhaps due to the stronger relative influence of precipitation that drove variation in the data. Insecticides and fungicides did not significantly impact growth or reproduction metrics at the subplot level.

**FIGURE 7:**
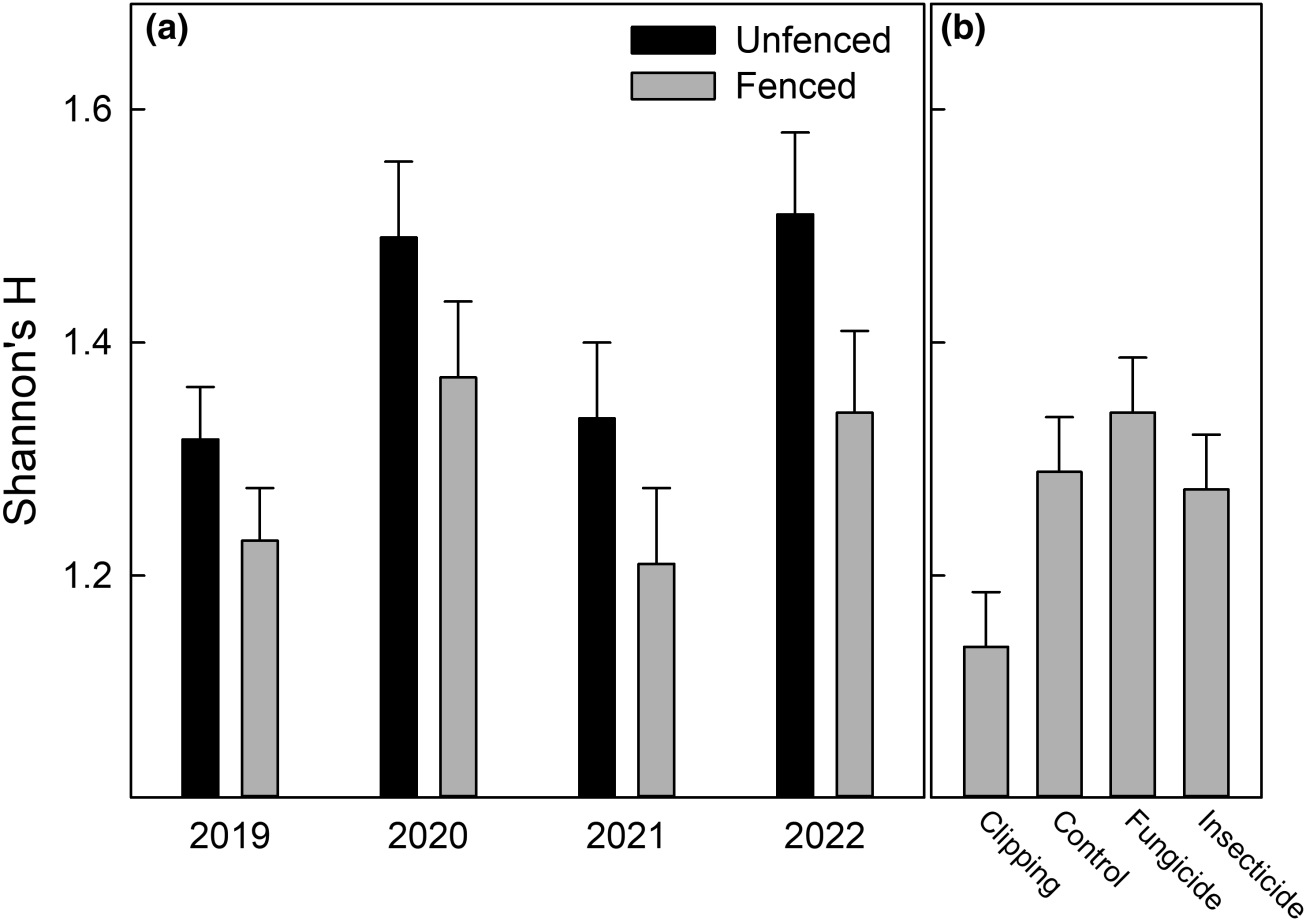
(a) The mean Shannon's H is shown as a function of fenced and unfenced plots across the study years. (b) The effect of the treatments is displayed on the same scale as panel (a). The error bars for each panel represent one standard error.

Ungulate herbivory and precipitation were associated with significantly higher Shannon's H (*F* = 12, *p* < .001, and *F* = 89, *p* < .001), respectively (Figure 7a). Further, ungulate herbivory and precipitation interacted such that the positive impact of ungulate herbivory was increased with higher precipitation (*F* = 17, *p* < .001). Also, competition removal effectively reduced diversity (Figure 6b), as expected (*F* = 5.1, *p* < .001; Figure 5b). Model summaries are presented in Tables 1, 2, 3, 4.

**TABLE 1:** For basal area, the parameters are presented with their associated estimates, standard errors, degrees of freedom, *F*‐statistic, and *p*‐value.

Parameter	Estimate	SE	df	*F*‐value	*p*‐value
Basal area					
Intercept	5.14	0.20	1/1720	7600	<.001
Precipitation	0.002	0.0003	1/1720	18	<.001
Treatment					
Control	−0.54	0.18	3/1720	4.0	<.01
Fungicide	−0.38	0.18			
Insecticide	−0.51	0.17			
Year					
2020	−0.27	0.08	3/1720	12	<.001
2021	0.12	0.08			
2022	0.53	0.11			
Unfenced	−0.54	0.13	1/284	18.7	<.001
Location					
North	0.27	0.15	2/284	11.5	<.001
South	0.727	0.15			

**TABLE 2:** For height, the parameters are presented with their associated estimates, standard errors, degrees of freedom, *F*‐statistic, and *p*‐value.

Parameter	Estimate	SE	df	*F*‐value	*p*‐value
Height					
Intercept	35	2.1	1/1721	3070	<.001
Precipitation	0.06	0.005	1/1721	900	<.001
Year					
2020	−4.6	1.19	3/1721	88	<.001
2021	1.2	1.15			
2022	−14	1.13			
Unfenced	−27	1.68	1/284	303	<.001
Season(short)	−11	0.9	1/1721	136	<.001
Location					
North	2.1	1.5	2/284	2.2	.12
South	3	1.5			
Precipitation:Unfenced	0.02	0.004	1/1721	28	<.001

**TABLE 3:** For reproductive tillers, the parameters are presented with their associated estimates, standard errors, degrees of freedom, *F*‐statistic, and *p*‐value.

Parameter	Estimate	SE	df	*F*‐value	*p*‐value
R. Tillers					
Intercept	1.5	0.08	1/1719	670	<.001
Precipitation	0.00007	0.0002	1/1719	810	<.001
Treatment					
Control	−0.11	0.05	3/1719	1.9	.12
Fungicide	−0.10	0.05			
Insecticide	−0.10	0.05			
Year					
2020	0.71	0.08	3/1719	447	<.001
2021	−1.39	0.05			
2022	−0.11	0.08			
Unfenced	−0.13	0.036	1/284	13	<.001
Season(short)	0.35	0.05	1/1719	66	<.001
Location					
North	0.1	0.05	2/284	6.2	<.01
South	−0.06	0.04			

**TABLE 4:** For Shannon's H, the parameters are presented with their associated estimates, standard errors, degrees of freedom, *F*‐statistic, and *p*‐value.

Parameter	Estimate	SE	df	*F*‐value	*p*‐value
Shannon's H					
Intercept	1.29	0.06	1/1720	6250	<.001
Precipitation	−0.00004	0.00009	1/1720	89	<.001
Treatment					
Control	0.15	0.05	3/1720	5.1	<.01
Fungicide	0.16	0.05			
Insecticide	0.16	0.05			
Year					
2020	0.17	0.21	3/1720	36	<.001
2021	−0.01	0.02			
2022	0.14	0.02			
Unfenced	−0.011	0.04	1/284	12	<.001
Location					
North	−0.05	0.04	2/284	42	<.001
South	−0.36	0.04			
Precipitation:Unfenced	0.0004	0.00009	1/1720	17	<.001

**TABLE 5:** – For *Orseolia* sp. galls and *Tetramesa* sp. exit holes; the parameters are presented with their associated estimates, standard errors, degrees of freedom, *F*‐statistic, and *p*‐value.

Parameter	Estimate	SE	df	*F*‐value	*p*‐value
*Orseolia* galls					
Intercept	23	3.3			
Insecticide	−10	3.8	1/23	3.2	.09
Unfenced	−15	3.8	1/23	13	<.01
Location					
North	−8.8	3.3	2/23	5.1	.02
South	−9.5	3.3			
Insecticide:Unfenced	10	5.4	1/23	3.4	.08
*Tetramesa* exit holes					
Intercept	4.1	1.9			
Insecticide	2.8	2.2	1/23	0.15	.7
Unfenced	−1.0	2.2	1/23	4.5	.05
Location					
North	0.5	1.9	2/23	0.79	.47
South	−1.75	1.9			
Insecticide:Unfenced	−4.5	3	1/23	2.15	.16

### 3.3 Insect compensation in the absence of ungulates

**FIGURE 8:**
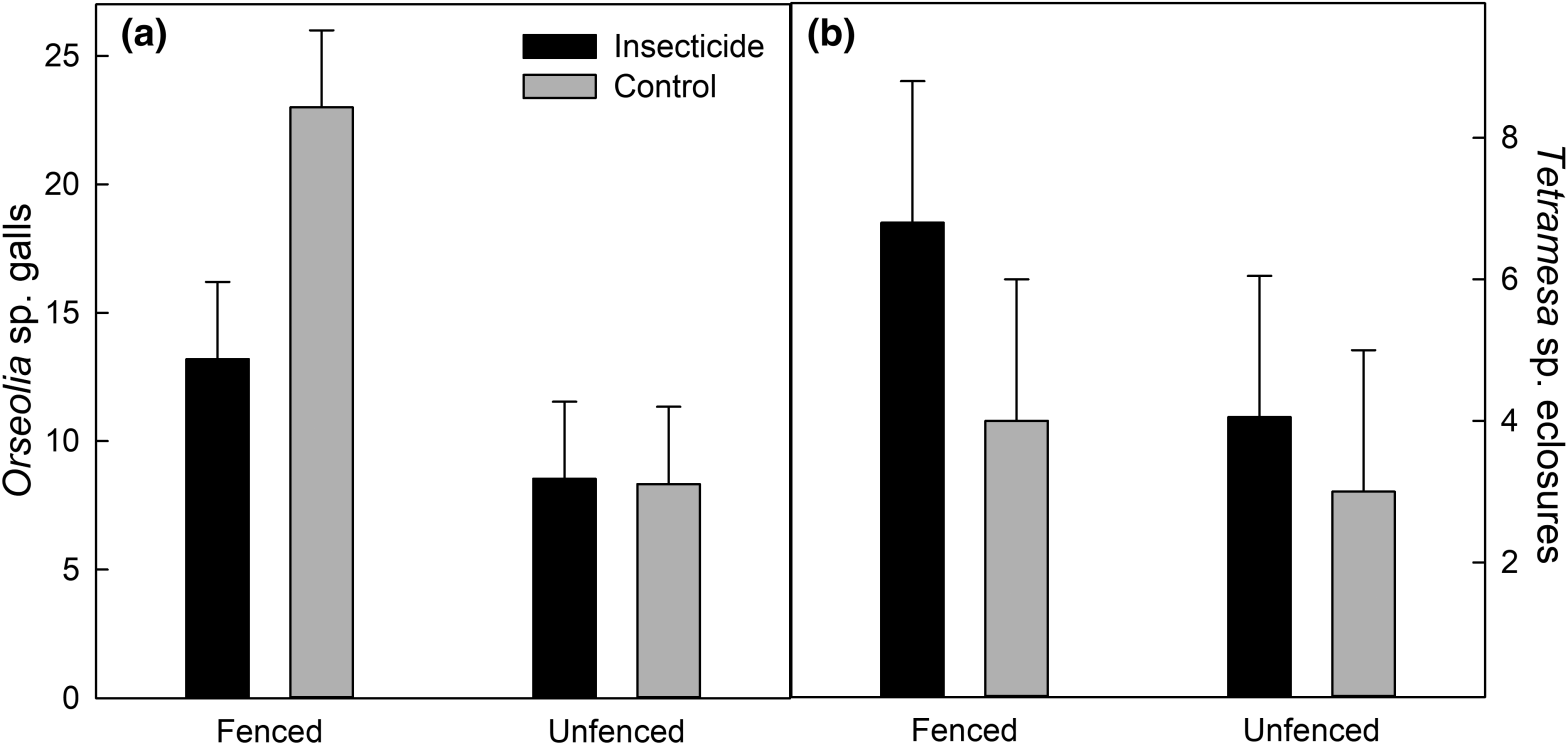
The number of Orseolia sp. galls, and Tetramesa sp. eclosure holes are shown on panels (a) and (b) *y* axes, respectively. The *y*‐axes have the same scale. Insecticide and control bars are grouped by fenced and unfenced treatments on the *x*‐axes. The error bar is one standard error.

Specialist insects increased when ungulates were excluded, revealing a potential compensatory shift of natural enemies. Ungulate exclusion increased *Orseolia* sp. galls in controls, but not in insecticide‐treated subplots, nearly doubling the galls in the fenced exclosures (*F* = 13, *p* = .002). Insecticide interacted with fencing to increase *Orseolia* sp. galls in treated and fenced plots, but insecticide did not increase galls in the presence of ungulate herbivory (*F* = 3.4, *p* = .08). Fencing was also associated with increased numbers of *Tetramesa* sp. exit holes but only by one more exit hole per approximately 50 tillers (*F* = 4.5, *p* < .05), and there was no significant interaction between fencing and insecticide use (Figure 8). This indicates a differential impact of ungulate herbivory on specialist insect herbivores. Model summaries are presented in Table 5.

### 3.4 Fungal smut outbreak

**FIGURE 9:**
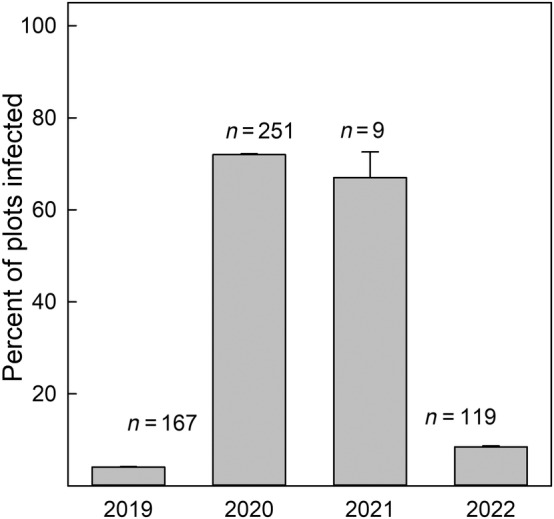
For each year, the percentage of plots with infected tussocks is shown. The total samples of tussocks with reproductive tillers evaluated are given above the bar for each year. 2021 had very few reproductive tillers produced. The error bars are one standard error.

Our smut observational study showed that broad outbreaks may follow regional weather patterns and could regulate buffelgrass reproduction at large spatial scales during an adverse weather event. The smut outbreak was associated with precipitation (*z* = 2.26, *p* = .024), year (*z* = 2.13, *p* = .033), and the interaction between six‐month lag precipitation and year (*z* = −1.84, *p* = .06). The probability of smut being present in a subplot was 4% in 2019, 73% in 2020, 67% in 2021, and 8% in 2022 (Figure 9). The smut specimen barcode sequence closely matched *Anthracocystis penniseti* (Rabenh.) McTaggart & R.G.Shivas (GBIF, 2023), indicating that this is a cosmopolitan smut species found on grass species belonging to *Cenchrus* and *Pennisetum* genera. The smut generally persisted at a low incidence rate in non‐outbreak years, and this outbreak was characterized by an above‐average wet period followed by a below‐average dry period.

## 4 DISCUSSION

Grass invasion remains a global issue that drives ecosystem degradation, alters grass‐fire cycle feedback, and is associated with globalization and human drivers. The complexities of invasion require integrated approaches to disentangle the influence of natural enemies and their interactions with each other and the abiotic environment. Fundamentally diverse hypotheses (Catford et al., 2009) which strive to understand biotic and abiotic interactions (Dawson et al., 2014; Heckman et al., 2017) in complex networks foreshadow the need for holistic understanding and integrated management (Harker & O'Donovan, 2013). We show that systematically removing these natural stressors approximates an invasion in buffelgrass' native range, revealing their hierarchical and interactive impacts. Detailed studies are needed to examine multiple interactions across resource gradients, focusing on plant–plant competition, resource availability, and natural enemies that independently or interactively influence plant invasion (Blumenthal et al., 2009; Heckman et al., 2017). Our study adds to this literature with a multifaceted approach that reveals a complex of natural enemies from diverse taxa that, with plant competition, negatively impacts buffelgrass along a precipitation gradient and differentially according to weather extremes.

Invasive organisms do not escape just one enemy or stressor. Evidence suggests that the quantity of enemies an organism escapes scales with its invasiveness (Mitchell & Power, 2003; Torchin et al., 2003) and that escape from natural stressors and direct and indirect competition interact with resource availability to promote invasion (Blumenthal et al., 2009; Mitchell et al., 2006). Examples have shown that reducing one group of natural enemies such as pathogens (Torchin et al., 2003), insect herbivores (Rhodes, Plowes, Martins, et al., 2022), ungulate herbivores (Rhodes, Plowes, Martins, et al., 2022), plant–plant competition (Lucero et al., 2020) or increased resource availability and its interaction with natural enemies (Blumenthal, 2005, 2006) increase the invasiveness of an organism. Combining these factors into a holistic framework of invasion ecology improves integrated invasive species management, especially in the face of variable precipitation accompanying climate change.

Climate change increases the frequency of extreme weather events (Cai et al., 2014), and the rate of extreme phases may increase nearly three times in future climate models (Cai et al., 2014). In addition, these phases spur various biological events such as phytoplankton blooms, locust, and malaria outbreaks (Hashizume et al., 2009; Todd et al., 2002). Our study was characterized by a long‐term drought impacting eastern Kenya driven by the Indian Ocean Dipole, drastically reducing precipitation during Northern Kenya's two regular rainy seasons (Blau & Ha, 2020). Generally, African C4 perennial invasive grasses are drought resilient (Williams & Baruch, 2000) and outcompete native plants during and after drought (Xu et al., 2017). High‐resource environments can benefit invasive species once released from natural enemies (Blumenthal et al., 2009). Release from natural enemies also benefits invasive plants in low‐resource environments (Heckman et al., 2017), suggesting that higher variability in resource availability may benefit invasive species in both high‐ and low‐resource environments characteristic of climate change. In introduced ranges, the competitive ability of drought and herbivore tolerance in successful invaders is generally higher than that of the native community (Ravi et al., 2022). For example, reducing herbivore impact can provide buffelgrass long‐term resilience to herbivory and drought due to root reserves (Rhodes et al., 2023). As such, natural enemies interacting with the abiotic environments may be fundamental to predicting invasiveness.

Our study captured a transition from a wet period to a historic drought, which showed strong interactive effects on buffelgrass productivity, especially when removing ungulate herbivory. The 2019 positive Indian Ocean Dipole was considered one of the most extreme in a century (Shi & Wang, 2021) and introduced high precipitation variability and was associated with buffelgrass' interaction with ungulates. Similarly, removing native ungulates was singularly implicated in defining the invasiveness of an exotic plant species in North America (Kalisz et al., 2014). Highly variable environments alter invasion (Ricciardi et al., 2021), and climate change often magnifies the impact of invasion (Ravi et al., 2022); some of that effect may be due to release from natural enemies. Ungulate herbivory is a critical driver of vegetation productivity and structure, but abiotic factors drive the magnitude of these impacts (Staver et al., 2021). Ungulates reduced buffelgrass basal area, height, and these impacts were muted in periods of high precipitation. Buffelgrass was less impacted by low precipitation regimes when protected from ungulate herbivory, similar to clipping experiments in its introduced range (Rhodes et al., 2023). Areas with higher precipitation in the southern portion of the Mpala Research Centre have increased vegetation productivity and show less of an impact from ungulate species (Goheen et al., 2018).

Consistent with buffelgrass in its introduced range, the increases in buffelgrass basal area lead to reductions in plant community diversity (Rhodes et al., 2021, 2023). Reduced buffelgrass cover in the presence of ungulate herbivory was associated with the increased diversity of the plant community. Diversity within fenced areas declined as highly competitive yet palatable buffelgrass increased abundance and reduced other plant species' abundances. These data suggest that apparent competition among the plant community under the pressure of a highly diverse group of ungulate herbivores contributes to plant community diversity and lowers the competitive ability of buffelgrass. This decrease in plant diversity when buffelgrass is present is similar to what has been observed in parts of buffelgrass' introduced ranges (Franklin et al., 2006; Smyth et al., 2009). Reductions in diversity have increasingly negative impacts on grassland communities and ecosystem function over time (Wagg et al., 2022). Conversely, plant–plant competition can alter invasion outcomes through biotic resistance (Lucero et al., 2020), and utilizing functional diversity can reduce the invasiveness of introduced grasses (Ammondt & Litton, 2012). Further, removing competition increased the buffelgrass‐dominated area within the subplot, which tended to increase reproductive output. This reduction suggests that propagule pressure increases when community diversity is low, potentially setting off an invasion cascade. Our results suggest that plant–plant competition in the native range is an important filter for buffelgrass dominance, which may escape these competitive effects in its introduced range.

The insecticide application had no significant impact on buffelgrass productivity or reproduction indicators—initially surprising but has several potential explanations. The local scale of insecticide application at the subplot level does not reduce the adult oviposition pressure on treated plants if the general insect population remains intact. After oviposition, the larvae may die, but some adverse effects on the plants may have already occurred. The use of systemic insecticides can alter the insect community dynamics greatly and make predictable responses difficult to capture (Cohen et al., 1994). Insecticides can lead to disorganization of the arthropod community, where increases in some insect populations correspond to reductions in others (Cohen et al., 1994). Given the highly diverse natural enemies in grass native ranges (Morrison, Plowes, et al., 2023; Rhodes, Plowes, Lawson, et al., 2022), reducing subsets of these guilds with insecticides and fungicides may not have sufficiently reduced the impact of these suites of organisms. Further, annual or seasonal fluctuations in insect communities introduce variability in plant damage from insects (Agrawal et al., 2005; Agrawal & Kotanen, 2003).

When we focused on two highly specific insect species *Orseolia* sp. and *Tetramesa* sp. (Morrison, Plowes, et al., 2023), we found that ungulate exclusion increased the number of insects feeding on buffelgrass. The mechanism is likely the removal of tillers for oviposition and development by ungulate grazing. This result suggests that when grasses in their native range lose a suite of natural enemies, there is some compensation from other functionally different natural enemies. These functionally different natural enemies compete, compensate, and interact with one another to have top‐down impacts on grass productivity (Agrawal et al., 2005). The insecticide treatment reduced *Orseolia* sp. but not *Tetramesa* sp. numbers, leading to differing impacts of insecticides across the species studied. The compensation by a group of natural enemies may be a recurrent theme in invasion ecology resulting from altered trophic cascades where ungulate herbivory may impact arthropods, small mammals, or reptiles (Goheen et al., 2018). For example, excluding large ungulate herbivores at experimental sites within Mpala Research Centre coincides with increased rodent density (Keesing, 1998), suggesting that further niche expansion across taxa is likely following the release from a dominant ecological stressor.

Systemic fungicide did not have long‐term impacts on buffelgrass area, height, or reproductive tillers. The use of fungicides or insecticides had little impact in a similar study (Heckman et al., 2016), which may suggest our chemical treatments were not broad enough or strong enough to sufficiently reduce these suites of abundant and diverse guilds of fungi. The commercially available fungicide we used was formulated to knock out oomycetes, a diverse group of organisms ubiquitous globally and often parasitic to plants (Thines & Kamoun, 2010). Of the oomycetes, the graminicolous downy mildews impact both wild and cultivated pasture grass in semi‐arid and sub‐tropics (Thines et al., 2008). However, our treatment through broad‐spectrum fungicide showed no patterns at the subplot level. In other studies, knocking out fungus with a broad‐spectrum fungicide showed no impact on plant recruitment or establishment (Heckman et al., 2017; Maron et al., 2013). Our nascent understanding of the fungal–plant interactions across complex networks (Dickie et al., 2017) may preclude nontargeted treatments of plants or soil with systemic fungicides. As research advances, the continuum of positive and negative fungal relationships with their grass hosts will help elucidate their role in invasion.

Our study captured a broad outbreak of smut fungi, regardless of ungulate or subplot treatment, being driven by weather patterns. Smut incidence was stable until a wet year followed a historic drought. Smut fungi are characterized by the infection of the host's reproductive tissues and the production of teliospores. Smuts that are often asymptomatic, living endophytically or in soils may have little year‐to‐year impact on grass productivity and reproduction (Zuo et al., 2019), yet when an outbreak occurs, their impact on the population and future generations are likely exponentially significant to future population dynamics (Farrell et al., 2002). Similarly, smut outbreaks of *Ustilago bullata* on *Bromus tectorum* followed weather patterns and landscape characteristics following fires that shifted disease incidence (Applestein et al., 2021). Our study showed similar associations with shifts from wet to dry periods. The teliospores of smut fungi allow survival in soils for years when moist, favorable conditions allow rapid proliferation. We observed favorable wet conditions in 2019 and 2020, with an abundance of reproductive tiller production in 2020 that coincided with the start of the outbreak as hot and dry conditions began. The Indian Ocean Dipole greatly impacts Kenya's weather and the biology of outbreaks of locusts and plankton in the region (Shi & Wang, 2021) and whether these shifts are related to smut outbreaks would be of interest as an episodic limiting factor in buffelgrass reproduction and dispersal (Meentemeyer et al., 2012). The reduction of diseases of buffelgrass may be influential in spreading unchecked in introduced ranges. Loss of pathogens has been well documented and is a classic case for the enemy release hypothesis (Jeschke, 2014; Mitchell & Power, 2003). The irruptive population behavior of some natural enemies deserves more attention, especially if they are associated with broad weather patterns. This would support a model for the establishment of semi‐arid grasses in the native range, where establishment may occur episodically in episodes of suitable weather but ahead of irruptive outbreaks of pathogens and insect herbivores that may have severe impacts on population growth. By extension, the establishment of these invasive species in introduced ranges may occur with fewer constraints from such natural enemies.

Buffelgrass remains a valued pasture grass for managed pastures, yet it negatively impacts ecosystem function and the biodiversity of flora and fauna when it escapes. This paradox has posed daunting challenges for ecosystem and wildlife management with the continued development of invasive grasses for land conversion (Ricciardi et al., 2021). Studying invasive grasses in their native range uncovers relevant stressors and a novel understanding of the ways natural enemies relate to invasive species through their interactions with the environment. For example, smut pathogens, if highly specific to buffelgrass, could reduce the spread of the grass to natural grasslands from managed pastures or rangelands yet not seriously reduce the forage production and value. Similarly, less conspicuous arthropod enemies attacking stem tissue or dead tissue may represent a compromise. A renewed interest in the biological control of grasses offers actionable targets (Sutton et al., 2019), and the development of frameworks for successful programs can lead to the restoration of invaded lands. Additionally, adaptive management of rangelands via targeted grazing effectively uses generalist ungulates as natural enemies and mitigates invasive plant impacts while maintaining multiple uses of livestock and wildlife (Rhodes et al., 2021). This study shows the importance of multiple stressors in defining the invasiveness of buffelgrass and how escape from a rich assemblage of herbivores, competitors, and pathogens may contribute to its global invasive success.

## AUTHOR CONTRIBUTIONS

**Aaron C. Rhodes:** Data curation (lead); formal analysis (lead); investigation (lead); methodology (equal); visualization (lead); writing – original draft (lead); writing – review and editing (equal). **Robert M. Plowes:** Conceptualization (lead); funding acquisition (lead); methodology (supporting); project administration (equal); resources (equal); supervision (equal); writing – review and editing (equal). **Elizabeth A. Bowman:** Conceptualization (equal); methodology (equal); writing – review and editing (equal). **Aimee Gaitho:** Data curation (equal); investigation (equal); project administration (equal); writing – review and editing (equal). **Ivy Ng'Iru:** Data curation (equal); investigation (equal); resources (equal); writing – review and editing (equal). **Dino J. Martins:** Funding acquisition (equal); project administration (equal); supervision (equal); writing – review and editing (equal). **Lawrence E. Gilbert:** Funding acquisition (equal); project administration (equal); supervision (equal); writing – review and editing (equal).

## FUNDING INFORMATION

The Lee and Ramona Bass Foundation provided funding for this research.

## CONFLICT OF INTEREST STATEMENT

The authors have no competing interests.

## Data Availability

Please find the raw data, R scripts, and annotations for this work at the Open Science Framework: (https://doi.org/10.17605/OSF.IO/MVD8U). Please contact the corresponding author for additional information on the data.
